# Investigating Tumour Responses to Combinations of Radiotherapy and Hyperthermia

**DOI:** 10.1007/s11538-025-01449-7

**Published:** 2025-07-01

**Authors:** Chloé Colson, Philip K. Maini, Helen M. Byrne

**Affiliations:** 1 Wolfson Centre for Mathematical Biology, Mathematical Institute, University of Oxford, Radcliffe Observatory Quarter, Oxford, OX2 6GG UK; 2 Centre for Evolution and Cancer, The Institute of Cancer Research, 15 Cotswold Rd, Sutton, SM2 5NG UK; 3 Nuffield Department of Medicine, Ludwig Institute for Cancer Research, University of Oxford, Roosevelt Drive, Oxford, OX3 7DQ UK

**Keywords:** Radiotherapy, Hyperthermia, Growth arrest mechanisms, ODE system

## Abstract

Hyperthermia (HT) is a promising candidate for enhancing the efficacy of radiotherapy (RT), but its use in the clinic has been limited by incomplete understanding of its interactions with RT. In this work, we investigate tumour responses to high temperature HT alone and combined with RT, focussing on how two different mechanisms for growth control may impact tumour sensitivity to these treatments. We extend an existing ordinary differential equation model of tumour growth and RT response to include high HT. In the absence of treatment, this model distinguishes between growth arrest due to nutrient insufficiency and competition for space, and exhibits three growth regimes: nutrient limited (NL), space limited (SL) and bistable (BS), where both mechanisms for growth arrest coexist. We construct three virtual tumour populations corresponding to the NL, SL and BS regimes and, for each population, we identify the treatment (RT, HT or RT + HT) and dosing regimen that maximise the reduction in tumour burden at the treatment end-point. We thus generate experimentally testable predictions that may explain highly variable experimental and clinical responses to RT and HT and assist patient-specific treatment design.

## Introduction

The efficacy of established cancer therapies, such as radiotherapy (RT) and chemotherapy, has been limited by various factors, including early termination due to intolerable treatment toxicity and tumour-specific treatment sensitivities. As a result, novel treatment combinations, which yield additional anti-cancer effects and mitigate treatment toxicity, have received increased focus. In particular, hyperthermia (HT), which involves heating tumour cells above physiological temperatures, has emerged as a potential candidate for increasing the efficacy of RT. However, the lack of a systematic investigation of the interactions between RT and HT and the mixed success of experimental and clinical studies combining them (Jha et al. 2016) have hampered the establishment of HT as an adjuvant therapy in the clinic. With cancer heterogeneity at phenotypic (and genetic) levels often being associated with treatment resistance (Marusyk et al. 2020), a better understanding of how intra- and inter-tumour heterogeneity may affect tumour sensitivity to RT and HT could help the development of effective patient-specific combination protocols.

In this paper, we extend an existing model of tumour growth and RT response (Colson et al. 2023) to investigate how nutrient and space limited mechanisms of growth control may influence tumour responses to combined RT and HT. We aim to offer possible explanations for the diverse reported experimental and clinical tumour responses, and guide the design of (personalised) treatment protocols by generating experimentally testable predictions about how RT and HT should be combined.

*Hyperthermia as a Potential Radiosensitiser*. HT involves heating tumour cells to temperatures in the range $39-50^{\circ }\hbox {C}$. The thermal dose is quantified by the treatment temperature and heating duration, and distinguishes mild and high HT. Mild HT typically refers to treatments at temperatures no higher than $42^{\circ }\,{\textrm{C}}$ for $30-60 \,\hbox {min}$. Evidence suggests it increases tumour oxygenation via increased tumour perfusion due to heat-induced vasodilation (Mueller-Klieser and Vaupel 1984), and/or reduced oxygen consumption rates following a switch from oxidative to glycolytic metabolism (Moon et al. 2010). Vasodilation fades within $1 \hbox {h}$ after heating (Vaupel and Kelleher 2010), while the reduction in oxygen consumption rates can persist for $24-48 \hbox {h}$ (Song et al. 2001). Mild HT may, thus, increase RT efficacy by reducing tumour hypoxia (i.e., oxygen insufficiency), which is known to promote radio resistance (Brizel et al. 1996, 1997).

High HT corresponds to heating at temperatures higher than $42^{\circ }\,{\textrm{C}}$ for $30-60 \, \hbox {min}$. It has three main effects: (1) it inflicts cytotoxic damage to cancer cells, largely through protein denaturation (Jung 1986; 2) it induces vascular stasis, damage and necrosis (Emami et al. 1980; Song 1984); and (3) it inhibits DNA repair pathways (Oei et al. 2015). Therefore, high HT may enhance tumour responses to RT by causing tumour and endothelial cell death, and also by inhibiting mechanisms responsible for RT-induced DNA damage repair (Maier et al. 2016).

There is no consensus on which of mild or high HT confers the greater radiosensitisation. Hence, we aim to compare the impact of mild and high HT on RT efficacy.

*Modelling Tumour Responses to RT and HT*. Many mathematical models have been developed to describe tumour responses to RT (Alfonso and Berk 2019; Celora et al. 2023; Enderling et al. 2010; Jeong et al. 2017; Lewin et al. 2018; Powathil et al. 2012; Prokopiou et al. 2015; Rockne et al. 2009) and HT (Brüningk et al. 2018b; Wright 2013) alone. These models typically assume that RT- and HT-induced cell death are instantaneous and can be described using probabilistic survival functions that are similar, or equivalent, to the classic Linear-Quadratic (LQ) model for RT cell death (McMahon and Prise 2019). The LQ model states that the fraction, *S*, of (tumour) cells that survive exposure to a dose *D* (${\textrm{Gy}}$) of radiation is given by

1 $$\begin{aligned} S(D) = e^{ - \left( \alpha D + \beta D^2\right) }, \end{aligned}$$

where $\alpha \ge 0$ and $\beta \ge 0$ are tissue-specific radiosensitivity parameters. The values of $\alpha$ and $\beta$ are usually inferred from cell survival data collected from *in vitro* tumour cell assays. While they capture the long-term amount of RT cell kill, they contain limited information about how the cell kill rate evolves during treatment, e.g., in response to combined treatment with an adjuvant modality such as HT. Since this limitation of the LQ model applies to all cell survival models, alternative models that describe cell death due to RT (Curtis 1986; Goodhead 1985; Neira et al. 2020; Scheidegger et al. 2013; Tobias 1985) and HT (O’Neill et al. 2011) as time-dependent processes have been proposed. By accounting for different types of damage (e.g., sub-lethal vs. lethal), damage repair and cell death in the case of insufficient repair or misrepair, such models keep track of the rate of cell death and the tumour composition (i.e., undamaged, damaged and dead cells) during treatment.

Significant work has also focussed on HT effects beyond cell kill. Spatially-resolved heat transfer models have been proposed to predict how variable tumour perfusion affects the time-dependent, intratumoural temperature distribution during HT (Attar et al. 2014; Bosque et al. 2022; Ezzat et al. 2014; Kumar and Rai 2016; Rai et al. 2020; Tunç et al. 2006). These models predict the intratumoural temperatures that can be achieved by different HT administration methods. When they account for HT cell kill, they assume first-order kinetics (e.g., the Arrhenius survival model) or an LQ-like survival model to minimise model complexity. The increase in tumour perfusion via vasodilation during mild HT has also been modelled (Bosque et al. 2021).

Very few mathematical models have been developed to study tumour responses to combined RT and HT. Existing approaches typically use the LQ model and modify the radiosensitivity parameters to account for the increase in cell death due to HT (Brüningk et al. 2018a; De Mendoza et al. 2021). While these temperature-dependent survival functions implicitly capture the thermal enhancement of RT response, they do not distinguish between the different ways in which HT may lead to radiosensitisation. They, thus, provide limited mechanistic insight into the interactions between HT and RT. By contrast, Scheidegger et al. (2013) investigated the effect of heat-induced inhibition of DNA repair, in particular, on tumour responses to RT by incorporating an ordinary differential equation (ODE) model of the time-dependent inactivation and activation of repair proteins by HT into a dynamic model of RT cell kill.

To the best of our knowledge, no existing mathematical model describes the range of mild and high HT effects previously discussed, alone or in combination with RT. We fill this gap in the literature by extending the dynamic model of tumour growth and time-dependent RT response developed in Colson et al. 2023 to include tumour responses to mild and high HT. In the absence of treatment, this model exhibits three distinct growth regimes: a nutrient limited (NL) regime, where tumours reach steady state when cell proliferation and cell death rates balance, a space limited (SL) regime, where tumours reach steady state when cell proliferation converges to zero, and a bistable (BS) regime, where both growth control mechanisms are simultaneously active (Colson et al. 2022). By distinguishing between different tumour growth patterns, the model can be used to investigate how they may explain treatment response heterogeneity. Indeed, we found that the model distinguishes between tumour responses to RT in each of the growth regimes (Colson et al. 2023). By extending it to also account for HT response, we can assess how nutrient and space limited mechanisms of growth arrest impact tumour responses to HT alone and combined with RT.

Our study shows that mild HT typically has a negligible effect on tumour responses to RT, across all growth regimes. Therefore, for brevity, the remainder of this paper focusses on tumour responses to high HT (${\mp }\,{\textrm{RT}}$). We refer the interested reader to Colson (2023) for results pertaining to mild HT response.

The remainder of the paper is structured as follows. In Sect. 2, we extend the ODE model of tumour growth and RT response in Colson et al. (2023) to include high HT. Using the methodology outlined in Sect. 3, we review the RT response of tumours in the NL, SL and BS growth regimes in Sect. 4. We then investigate their response to high HT in Sect. 5, and combined treatment in Sect. 6. We conclude in Sect. 7 by discussing our findings and outlining possible avenues for future work.

## Model Development

**Fig. 1:**
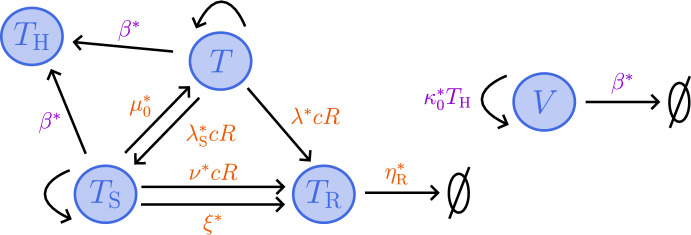
Schematic showing the interactions between *T*, $T_{\text {S}}$, $T_{\text {R}}$ and $T_{\text {H}}$ cells, the vasculature, *V*, and the oxygen concentration, *c*, in response to high HT (purple) and RT (orange) in the model (2)–(7).

In this section, we present the model of tumour growth and RT response developed in Colson et al. (2023) and extend it to include tumour responses to high HT. We denote by *T*(*t*), $T_{\text {S}}(t)$, $T_{\text {R}}(t)$ and $T_{\text {H}}(t)$, respectively, the undamaged, sub-lethally RT-damaged, lethally RT-damaged and lethally HT-damaged tumour cell volumes, by *c*(*t*) the intratumoural oxygen concentration, and by *V*(*t*) the vascular volume at time *t*. Letting $\Sigma = T+T_{\text {S}}+T_{\text {R}}+T_{\text {H}}+V$ be the total tumour volume and *R*(*t*) be the radiation dose rate, we propose the following system of time-dependent ODEs to describe tumour responses to treatments comprising RT and HT (see also the schematic in Fig. 1):

2 $$\begin{aligned} &  \frac{{\hbox {d}}{T}}{{\hbox {d}}{t}} = {q_2^* c T (S_{\text {max}} - \Sigma )} - \big (\delta ^*(c^*_{\text {min}} - c)H(c^*_{\text {min}}-c) + \lambda ^* c R + \nu ^* c R\big )T \nonumber \\ &  \qquad \qquad + \mu ^*(t) T_{\text {S}} - {\underbrace{ \beta ^*(t)T,}_\text {{high HT damage}}} \end{aligned}$$

3 $$\begin{aligned} &  \frac{{\hbox {d}}{T_{\text {S}}}}{{\hbox {d}}{t}} = {{\theta _2 q^*_2 } c T_{\text {S}}(S_{\text {max}} - \Sigma )} - \big (\delta _{\text {S}}^*(c^*_{\text {min}} - c)H(c^*_{\text {min}}-c) + \lambda ^*_{\text {S}} c R + \mu ^*(t) +\xi ^* \big )T_{\text {S}} \nonumber \\ &  \qquad \qquad +{\nu ^*c R T} -{\underbrace{ \beta ^*(t)T_{\text {S}},}_\text {{high HT damage}}} \end{aligned}$$

4 $$\begin{aligned} &  \frac{{\hbox {d}}{T_{\text {R}}}}{{\hbox {d}}{t}} ={\lambda ^* c R T} +{(\xi ^*+\lambda ^*_{\text {S}} c R) T_{\text {S}}}- \eta ^*_{\text {R}} T_{\text {R}}, \end{aligned}$$

5 $$\begin{aligned} &  {\frac{{\hbox {d}}{T_{\text {H}}}}{{\hbox {d}}{t}} = \beta ^*(t) (T+T_{\text {S}}) - \underbrace{{\eta ^*_{\text {H}}}T_{\text {H}},}_\text {{clearance}}} \end{aligned}$$

6 $$\begin{aligned} &  { \frac{{\hbox {d}}{V}}{{\hbox {d}}{t}} = \underbrace{\kappa ^*_0 T_{\text {H}}}_\text {{damage-induced angiogenesis }} - \underbrace{ \beta ^*(t)V,}_\text {{high HT damage}} } \end{aligned}$$

7 $$\begin{aligned} &  \frac{{\hbox {d}}{c}}{{\hbox {d}}{t}} = g^*(c^*_{\text {max}}-c) V - (q^*_1 c (T + \theta _1 T_{\text {S}}) + q^*_3 c(S_{\text {max}} - \Sigma ) \left( T+\theta _2 T_{\text {S}}\right) ), \end{aligned}$$

where *H*(*x*) denotes the Heaviside step function:

8 $$\begin{aligned} H(x) = {\left\{ \begin{array}{ll} 1, & \quad \text {if } x \ge 0, \\ 0, & \quad \text {if } x < 0, \end{array}\right. } \end{aligned}$$

and we impose the following initial conditions:

9 $$\begin{aligned} \Sigma (0) = T(0) + V(0) \in [0,S_{\text {max}}], \quad T_{\text {S}}(0) = T_{\text {R}}(0) = T_{\text {H}}(0) = 0, \quad c(0) \in [0,c^*_{\text {max}}]. \nonumber \\ \end{aligned}$$

In (9), we assume that no cells are damaged prior to the start of treatment. We set $0 \le \Sigma (0) \le S_{\text {max}}$ and $0 \le c(0) \le c^*_{\text {max}}$ to ensure physically realistic solutions for $t \ge 0$.

*Modelling Tumour Growth*. Setting $R \equiv 0$ and $\beta ^* \equiv 0$ in Eqs. (2)–(7), we have ${T_{\text {S}} \equiv T_{\text {R}} \equiv T_{\text {H}} \equiv 0}$ and $V \equiv V(0)$, and we recover the model of tumour growth developed in Colson et al. (2022). Letting $S_{\text {max}}$ be the total available volume, we suppose that undamaged tumour cells, *T*, proliferate at a rate proportional to the total free volume, $(S_{\text {max}} - \Sigma )$, and the oxygen concentration, *c*, with proportionality constant $q_2^* > 0$. If $0 \le c < c^*_{\text {min}}$, the hypoxic oxygen threshold, then tumour cells die at a rate proportional to $(c^*_{\text {min}}-c)$, with proportionality constant $\delta ^*>0$. We assume further that oxygen is supplied to the tumour at a rate proportional to *V*(0) and $(c^*_{\text {max}}-c)$, where $c^*_{\text {max}} > 0$ represents the constant oxygen concentration in the vasculature. Finally, undamaged tumour cells consume oxygen for maintenance at a rate proportional to *c*, with proportionality constant $q^*_1>0$, and also for proliferation at a rate proportional to their proliferation rate, with conversion factor $k^*> 0$ such that $q^*_{3} = \frac{q^*_{2}}{k^*}$.

*Modelling Radiotherapy Response*. Letting $R \ge 0$ and setting $\beta ^* \equiv 0$ in Eqs. (2)–(7), we have $T_{\text {H}} \equiv 0$ and $V \equiv V(0)$, and we recover the model of RT response proposed in Colson et al. (2023). We assume that the proliferation, oxygen consumption and death of undamaged tumour cells, *T*, are unchanged by RT. Assuming further that the tumour is exposed to a total radiation dose $D \, ({\text {Gy}})$ during the time period $t_{\text {R}} \le t \le t_{\text {R}}+\delta _{\text {R}}$, measured in minutes, we define the constant RT dose rate *R*(*t*) as

10 $$\begin{aligned} R(t) = {\left\{ \begin{array}{ll} D/\delta _{\text {R}}, & \quad \text {if} \, \, t_{\text {R}} \le t \le t_{\text {R}}+\delta _{\text {R}},\\ 0, & \quad \text {otherwise}.\end{array}\right. } \end{aligned}$$

During RT, *T* cells suffer sub-lethal and lethal damage at rates proportional to the oxygen concentration, *c*, and the RT dose rate, *R*, with proportionality constants $\nu ^*>0$ and $\lambda ^*>0$, respectively. Sub-lethal damage is repaired at a constant rate $\mu _0^* > 0$ while un-repaired damage causes tumour cell death in two ways: (1) as it accumulates, the damage becomes lethal at a rate proportional to *c* and *R*, with proportionality constant, $\lambda _{\text {S}}^*>0$, and (2) sub-lethally damaged cells, $T_{\text {S}}$, undergo mitotic catastrophe (MC) if they attempt to divide with DNA damage. For simplicity, we assume MC occurs at a constant rate $\xi ^*>0$.

Like undamaged tumour cells, $T_{\text {S}}$ cells proliferate, consume oxygen (for proliferation and maintenance) and die. They proliferate at a rate proportional to both $(S_{\text {max}}-\Sigma )$ and *c*, with proportionality constant $q^*_{2,\text {S}} = \theta _2q^*_2$, where $\theta _2 \in (0,1)$. Thus, sub-lethally damaged cells proliferate slower than undamaged cells while they repair their damage. Accordingly, they consume oxygen for maintenance at a rate proportional to *c*, with proportionality constant $q^*_{1,\text {S}} = \theta _1 q^*_1$, where $\theta _1 > 1$ to indicate the greater oxygen demands of sub-lethally damaged cells for DNA repair. Further, $T_{\text {S}}$ cells consume oxygen for proliferation at a rate proportional to their proliferation rate so that $q^*_{3,\text {S}} = q^*_{2,\text {S}}/k^*$, where we have used the same conversion factor as for *T* cells for simplicity. Lastly, when $0 \le c < c^*_{\text {min}}$, $T_{\text {S}}$ cells die due to nutrient limitations at a rate proportional to $(c-c^*_{\text{ min }})$, with proportionality constant $\delta _{\text {S}}^*>0$.

Finally, $T_{\text {R}}$ cells cannot repair their damage and do not consume oxygen or proliferate. However, they occupy space and are degraded at a constant rate $\eta ^*_{\text {R}} > 0$.

*Modelling High HT Response*. We assume that high HT irreversibly damages viable tumour cells (*T*, $T_{\text {S}}$) and endothelial cells (*V*) at a constant rate ${\widetilde{\beta }} > 0$ during the heating period $t_{\text {H}} \le t \le t_{\text {H}} + \delta _{\text {H}}$, measured in minutes, so that

11 $$\begin{aligned} \beta ^*(t) = {\left\{ \begin{array}{ll} {\widetilde{\beta }}, & \quad \text {if } t_{\text {H}} \le t \le t_{\text {H}} + \delta _{\text {H}}, \\ 0, & \quad \text {otherwise}. \end{array}\right. } \end{aligned}$$

**Fig. 2:**
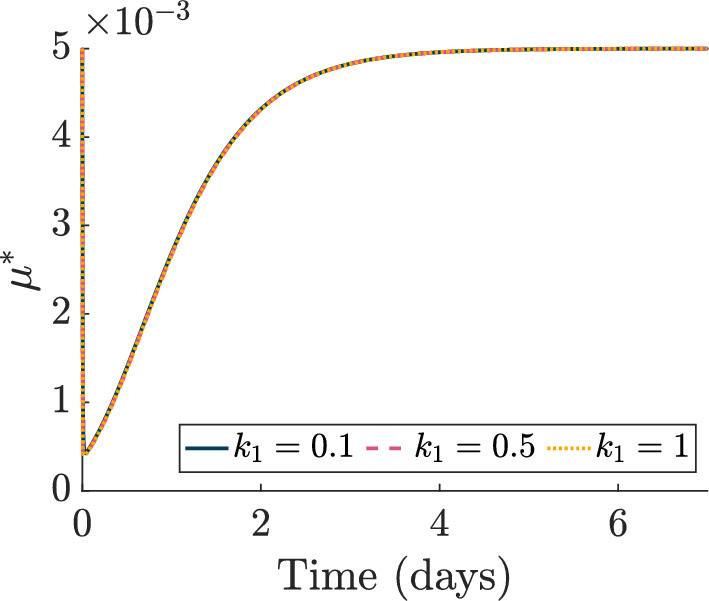
Given a high HT treatment of duration $\delta _{\text {H}} = 60 \,\hbox {min}$ starting at time $t_{\text {H}} = 0$, we plot $\mu ^*(t)$, defined in Eq. (13), for $t \in [0,7] \, \hbox {days}$. The parameters $\mu _0=\mu _0^*\tau$, with $\tau = 1 \,\hbox {min}$, $k_2$ and $\mu _\Lambda$ are fixed to the values stated in Table 2, $k_1 = 100 {\widetilde{\beta }}$ and ${\widetilde{\beta }} \in \{ 0.001, 0.005, 0.01\}$. There is no significant difference in the time evolution of $\mu ^*$ as $k_1$ varies. Thus, in this parameter regime, the inhibition of DNA repair is treatment-independent.

As heat-induced cell death occurs more slowly than heat-induced vascular stasis and necrosis (Song et al. 1980), we assume that heat-damaged cells, $T_{\text {H}}$, are degraded at a constant rate $\eta ^*_{\text {H}} > 0$ while dead vascular material is immediately cleared from the system. We also suppose that $T_{\text {H}}$ cells promote vascular growth at a rate proportional to their volume $(T_\textrm{H})$, with proportionality constant $\kappa ^*_0 > 0$ (Kanamori et al. 1999). Finally, we account for the inhibition of DNA repair under high HT by assuming that the RT-damage repair rate depends on exposure to high HT. Following Scheidegger et al. (2013), we suppose that, after a single dose of high HT, the ratio of inactive repair proteins to the total amount of repair proteins evolves as follows:

12 $$\begin{aligned} \Lambda (t) = {\left\{ \begin{array}{ll} 0, & \quad \ \text {if } 0 \le t< t_{\text {H}}, \\ \frac{k_1}{k_1+k_2} \left( 1-e^{-(k_1+k_2)(t-t_{\text {H}})}\right) , & \quad \text { if } t_{\text {H}} \le t < t_{\text {H}} +\delta _{\text {H}}, \\ \frac{k_1}{k_1+k_2} \left( 1-e^{-(k_1+k_2)\delta _{\text {H}}}\right) e^{-k_2(t-(t_{\text {H}}+\delta _{\text {H}}))}, & \quad \text { if } t \ge t_{\text {H}} + \delta _{\text {H}}, \end{array}\right. } \end{aligned}$$

where the positive constants $k_1$ and $k_2$ are the rates of inactivation and reactivation of repair proteins, respectively. Then, the time-dependent repair rate $\mu ^*(t)$ is defined as

13 $$\begin{aligned} \mu ^*(t) = \mu ^*_0 e^{-\mu _\Lambda \Lambda (t)}, \end{aligned}$$

where $\mu ^*_0 > 0$ is the repair rate in the absence of high HT (as defined above) and $\mu _\Lambda > 0$ represents the extent to which high HT inhibits DNA repair. Figure 2 shows that $\mu ^*(t), \, t \ge 0,$ is independent of the values of $k_1$ considered in our study, given fixed values of $\mu ^*_0$, $k_2$ and $\mu _\Lambda$ (see Table 2). The inter-tumour differences in high HT efficacy discussed in Sects. 5 and 6 are, therefore, attributable to the tumour and endothelial cell kill effects of high HT. The present study focusses on these two effects as our simulation results suggest they have a greater impact on high HT response than DNA repair inhibition (results not shown).

### Non-dimensionalisation

We non-dimensionalise Eqs. (2)–(7) and the initial conditions (9) by introducing the following scalings:

$$\begin{aligned}&{\widehat{T}} = \frac{T}{S_{\text {max}}}, \quad {\widehat{T}}_{\text {S}} = \frac{T_{\text {S}}}{S_{\text {max}}}, \quad {\widehat{T}}_{\text {H}} = \frac{T_{\text {H}}}{S_{\text {max}}}, \quad {\widehat{T}}_{\text {R}} = \frac{T_{\text {R}}}{S_{\text {max}}},\quad \\&{\widehat{V}}=\frac{V}{S_{\text {max}}}, \quad {\widehat{S}} = \frac{S}{S_{\text {max}}}, \quad {\widehat{c}}= \frac{c}{c_{\text {max}}}, \quad {\widehat{R}}= \frac{R}{R_{\text {max}}}, \quad {\widehat{t}} = \frac{t}{\tau }. \end{aligned}$$

We seek to capture interactions between the time-dependent effects of RT (tumour cell damage and repair) and HT (tumour cell and vascular death, and inhibition of DNA repair mechanisms). Since these effects operate on timescales of minutes to days, we set the timescale of interest to be $\tau = 1 \, \hbox {min}$. The maximum dose rate is also fixed at $R_{\text {max}} = 1 \, {\text {Gy}}/{\text {min}}$ (Konopacka et al. 2016). Under these scalings, and dropping hats for notational convenience, we obtain the following dimensionless system:

14 $$\begin{aligned} &  \frac{{\hbox {d}}{T}}{{\hbox {d}}{t}} = q_2 c(1 - \Sigma )T - \big (\delta (c_{\text {min}} - c)H(c_{\text {min}}-c) + \lambda c R + \nu c R \big )T \nonumber \\ &  \qquad \qquad + \mu (t)T_{\text {S}} - \beta (t)T, \end{aligned}$$

15 $$\begin{aligned} &  \frac{{\hbox {d}}{T_{\text {S}}}}{{\hbox {d}}{t}} = \theta _2 q_2 c (1 - \Sigma )T_{\text {S}} - \big (\delta _{\text {S}}(c_{\text {min}} - c)H(c_{\text {min}}-c) + \lambda _{\text {S}} c R + \mu (t) + \xi \big )T_{\text {S}} \nonumber \\ &  \qquad \qquad +{\nu R cT - \beta (t)T_{\text {S}}}, \end{aligned}$$

16 $$\begin{aligned} &  \frac{{\hbox {d}}{T_{\text {R}}}}{{\hbox {d}}{t}} ={\lambda c R T} +{(\lambda _{\text {S}} c R + \xi ) T_{\text {S}}}- \eta _{\text {R}} T_{\text {R}}, \end{aligned}$$

17 $$\begin{aligned} &  \frac{{\hbox {d}}{T_{\text {H}}}}{{\hbox {d}}{t}} ={\beta (t)} (T+T_{\text {S}}) - {\eta _{\text {H}}}T_{\text {H}}, \end{aligned}$$

18 $$\begin{aligned} &  \frac{{\hbox {d}}{V}}{{\hbox {d}}{t}} = \kappa _0 T_{\text {H}} - \beta (t) V, \end{aligned}$$

19 $$\begin{aligned} &  \frac{{\hbox {d}}{c}}{{\hbox {d}}{t}} =g(1-c)V - \left( q_1 c (T + \theta _1 T_{\text {S}}) + q_3 c(1- \Sigma ) \left( T+\theta _2 T_{\text {S}}\right) \right) , \end{aligned}$$

where

20 $$\begin{aligned} \begin{aligned}&{q_1}= {q_1^* S_{\text{ max }} \tau },\; {q_3}={q_3^* S_{\text{ max }} \tau }, \; {q_2}= q_{2}^* S_{\text{ max }} c^*_{\text{ max }} \tau , \; k = \frac{c^*_{\text{ max }}}{S_{\text{ max }}}k^*, \\ &c_{\text{ min }} = \frac{c^*_{\text{ min }}}{c^*_{\text{ max }}}, \; \delta = {\delta ^* c^*_{\text{ max }}\tau }, \; \delta _{\text{ S }}= {\delta _{\text{ S }}^*c^*_{\text{ max }} \tau }, \; g = {g^* S_{\text{ max }} \tau },\\ &\lambda = {\lambda ^* c^*_{\text{ max }} R_{\text{ max }} \tau }, \; \lambda _{\text{ S }}= {\lambda _{\text{ S }}^* c^*_{\text{ max }} R_{\text{ max }} \tau }, \; \nu = {\nu ^* c^*_{\text{ max }} R_{\text{ max }} \tau }, \\ &\beta (t) = {\beta ^*(t) \tau }, \; \kappa _0 ={\kappa _0^* \tau }, \; \mu (t) ={\mu ^*(t) \tau }, \; \xi = {\xi ^* \tau }, \; \eta _{\text{ H }} = {\eta _{\text{ H }}^* \tau }, \; \eta _{\text{ R }} = {\eta _{\text{ R }}^* \tau },\end{aligned} \end{aligned}$$

and subject to the initial conditions

21 $$\begin{aligned} \Sigma (0) = T(0)+V(0) \in [0,1], \quad T_{\text {S}}(0) = T_{\text {R}}(0) = T_{\text {H}}(0) = 0, \quad c(0) \in [0,1]. \nonumber \\ \end{aligned}$$

## Methods

We aim to characterise tumour responses to high HT in the nutrient limited (NL), space limited (SL) and bistable (BS) regimes exhibited by our model, and then to investigate whether combining treatments enhances tumour responses compared to high HT or RT in these regimes. To do so, we retrieve the NL, SL and BS virtual tumour populations constructed in Colson et al. (2023) (Sect. 3.1) and define a range of RT, HT and ${\text {RT}}+{\text {HT}}$ fractionation schedules (Sect. 3.2). Our methods for studying high HT responses and comparing tumour responses to the three different treatments are then described in Sects. 3.3 and 3.4, respectively.

### Three Virtual Tumour Populations

To construct the NL, SL and BS virtual tumour populations introduced in Colson et al. (2023), we fixed all tumour growth model parameters, except $q_1$, $q_3$ and *V*(0), at the default values stated in Table 2. We fixed $V(0) = 0.0005$ (NL), $V(0) = 0.005$ (SL) and $V(0) = 0.00275$ (BS); note that, in the absence of high HT, the vascular volume in each cohort is fixed at these values. We then generated three virtual tumour populations of size $N=250$ by randomly selecting $(q_3,q_1)$ pairs, corresponding to the NL, SL and BS regimes, respectively, from the regime-specific uniform distributions defined in Table 1.

**Table 1:** Uniform distributions *U*(*a*, *b*) used to sample the pairs of $q_1$ and $q_3$ values that correspond to the NL, SL and BS cohorts, respectively. For *V*(0) fixed, $\bar{q_1}$ is the threshold value above which only NL steady states exist, while, for *V*(0) and $q_1 \le \bar{q_1}$ fixed, $\bar{q_3}$ is the threshold value above which both NL and SL steady states co-exist and below which only SL steady states exist. Analytic expressions for $\bar{q_1}$ and $\bar{q_3}$ are presented in Appendix B of Colson et al. (2023)

Cohort	NL	SL	BS
Distribution used to sample $q_1$	$U(\bar{q_1},10)$	$U(0.01,\bar{q_1})$	$U(0.01,\bar{q_1})$
Distribution used to sample $q_3$	*U*(0.01, 10)	$U(0.01,\bar{q_3})$	$U(\bar{q_3},10)$

**Fig. 3:**
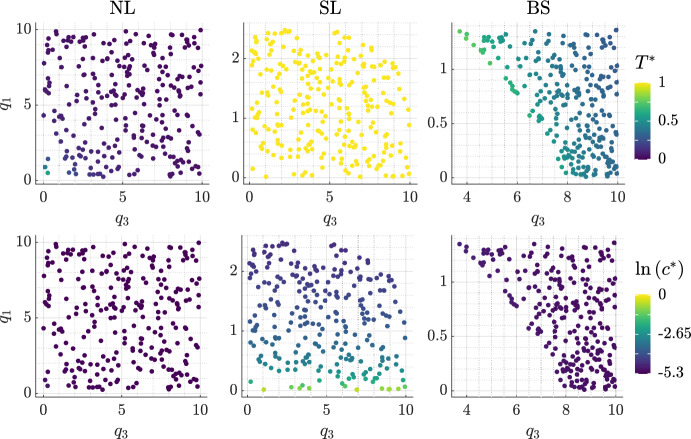
Scatter plots showing the steady state tumour volume, $T^*$, and logarithm of the oxygen concentration, $\ln {(c^*)}$, in the absence of treatment for each $(q_3,q_1)$ pair and fixed value of *V*(0) used to generate the virtual NL, SL and BS tumours. We show the NL steady states for the BS cohort; its SL steady states are qualitatively the same as those for the SL cohort, except ${T^*:= 1-V(0)=0.99725}$ and $\ln {(c^*)} \in [-4.6, -0.55]$.

Figure 3 shows the steady state tumour volume, $T^*$, and logarithm of the oxygen concentration, $\ln {(c^*)}$, in the absence of treatment for the $(q_3,q_1)$ pairs that define each cohort. Tumours in the NL cohort have the smallest values of $T^*$ and $c^*$, while tumours in the SL cohort have the largest values. In the NL and BS cohorts, $T^*$ decreases as $q_1$ and $q_3$ increase, whereas ${T^*:= 1-V(0) = 0.995}$ for all tumours in the SL cohort. The variability in $c^*$ is low in the NL and BS cohorts, while $c^*$ decreases significantly as $q_1$ increases in the SL cohort.

### The Treatment Schedules Under Consideration

Combined treatment dosing regimens vary considerably across clinical trials (Cihoric et al. 2015). In many cases, information about study design and treatment protocol is incomplete. Most RT schedules are designed in a standard way: a maximum tolerated dose (MTD) in the range $24-100\, {\text {Gy}}$ is fixed and radiation doses in the range $1-5\, {\text {Gy}}$ are delivered $1-5$ times per week. HT is also fractionated, and being applied $1-3$ times per week, at minimum temperatures in the range $42-45^{\circ }\hbox {C}$ for $30-120 \, \hbox {min}$, for high HT. The total number of HT fractions is determined by the length of the RT schedule (e.g., 5 weeks of RT is combined with 5 weeks of HT) or fixed such that HT is applied for a certain number of weeks at the start of treatment. In particular, a MTD for HT has not been reported. Further, HT is typically applied $30-240 \, \hbox {min}$ after RT.

We consider the following combined treatments. During combined treatment, $N_{\text {frac}}$ weekly RT fractions, of duration $\delta _{\text {R}} = 10 \, \hbox {min}$ and dose $D \, \hbox {Gy}$, are administered at the same time each day. The first weekly RT fraction is applied on Mondays and subsequent RT fractions are applied at equally spaced time intervals during Monday to Friday. One weekly dose of HT of duration $\delta _{\text {H}} = 60 \, \hbox {min}$ is also applied on Mondays, immediately after the RT fraction. Thus, $t_{\text {H}} = t_{\text {R}} + \delta _{\text {R}} + 1$, where $t_{\text {H}}$ and $t_{\text {R}}$ are the most recent times HT and RT were applied, respectively. While combined treatment is not significantly influenced by the order of, or the delay between, RT and HT (results not shown), we assume a minimal delay between RT and HT as this maximises HT-induced radiosensitisation *in vitro* (Mei et al. 2020).

We define the RT schedules as in Colson et al. (2023) by varying the dose amount, $D \in \llbracket 0,5 \rrbracket \, \hbox {Gy}$, and the number of weekly RT fractions, $N_{\text {frac}} \in \{1,3,5\}$. We impose a MTD, $D_{\text {max}} = 80 \, \hbox {Gy}$, for RT based on reported MTDs for different solid tumours (Pahlajani et al. 2012; Rosenzweig et al. 2005). The duration of each combined treatment, $N_{\text {wks}}$ (weeks), is fixed so that the total radiation dose administered is $D_{\text {max}}$ (or the closest multiple of *D* to $D_{\text {max}}$). We also impose a MTD, $\beta _{\text {max}}$, for HT, and vary the thermal dose ${\widetilde{\beta }} \in \{0.001, 0.003, 0.005, 0.007, 0.009,0.01 \}$ such that ${\widetilde{\beta }}N_{\text {wks}} \le \beta _{\text {max}}$. Since no MTD has been reported for HT, we fix $\beta _{\text {max}} = 0.08$ based on preliminary numerical simulations showing that a total HT dose ${\widetilde{\beta }}N_{\text {wks}} \ge 0.8$ typically leads to complete tumour eradication, while HT typically has a negligible effect for ${\widetilde{\beta }}N_{\text {wks}} \le 0.0008$ (results not shown). Fixing $\beta _{\text {max}} = 0.08$, we observe a physically realistic range of tumour responses to HT.

**Fig. 4:**
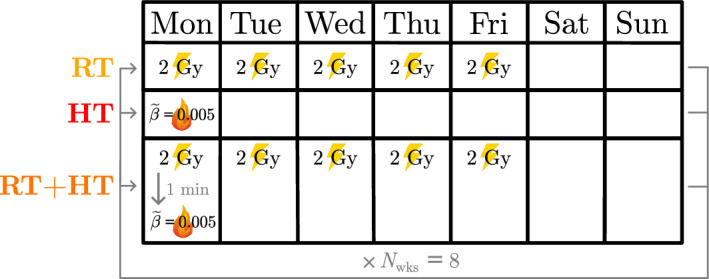
Schematic showing the RT, HT and ${\text {RT}}+{\text {HT}}$ fractionation schedules whose efficacy we compare when combining a conventional RT schedule comprising $5\times 2 \, \hbox {Gy}$ for $N_\text {wks} = 8$ weeks and a conventional HT schedule with ${\widetilde{\beta }} = 0.005$. Yellow lightning symbols represent a RT fraction and red flame symbols represent a high HT fraction.

We compare each combined treatment to treatment with RT alone (${\widetilde{\beta }} \equiv 0$) and HT alone ($R \equiv 0$); see Fig. 4.

### Numerical Methods

For each virtual tumour and each treatment (RT, HT or ${\text {RT}}+{\text {HT}}$) protocol, we solve Eqs. (14)–(19) numerically for $t \in (0,t_{\text {end}}]$, where $t_{\text {end}}$ is the treatment end-point. We use MATLAB’s ODE45, a single step built-in solver for non-stiff ODEs based on an explicit Runge–Kutta (4,5) formula, the Dormand-Prince pair (Dormand and Prince 1980). For simplicity, we impose the initial conditions

22 $$\begin{aligned} \big (T(0), T_{\text{ S }}(0), T_{\text{ R }}(0), T_{\text{ H }}(0), V(0),c(0) \big ) = \big (T^*, 0,0, 0, V(0),c^* \big ), \end{aligned}$$

where $T^*$ and $c^*$ are, respectively, the steady state tumour volume and oxygen concentration in the absence of treatment (recall Fig. 3).

**Table 2:** List of dimensionless tumour growth, RT response and high HT response parameters and values assigned to them for the purpose of the model analysis and numerical simulations

Model	Parameters	Definition	Value(s)
Growth	$c_{\text {min}}$	Anoxic oxygen threshold	$10^{-2}$
	*g*	Rate of oxygen exchange per unit vascular volume	5
	*k*	Conversation factor	$10^{-2}$
	$$q_1$$	$${\text {O}}_2$$ consumption rate for maintenance	$$ [10^{-2}, 10]$$
	$\theta _1$	Proportionality constant relating $q_1$ and $q_{1,S}$	10
	$$q_3$$	$${\text {O}}_2$$ consumption rate for proliferation	$$ [10^{-2}, 10]$$
	$$q_{2} $$	Proliferation rate	$$ k_{qs}$$
	$\theta _2$	Proportionality constant relating $q_2$ ($q_3$) and $q_{\text {2,S}}$ ($q_{\text {3,S}}$)	$10^{-1}$
	$\delta ,\delta _{\text {S}}$	Rates of death due to nutrient insufficiency	$q_2, \theta _2 q_2$
	*V*(0)	Vascular volume	$(0,5 \times 10^{-3}]$
RT	$\nu$	RT sub-lethal damage rate	10
	$\lambda , \lambda _{\text {S}}$	RT lethal damage rates	1
	$\mu _0$	Repair rate constant	$5\times 10^{-3}$
	$\zeta$	Rate of death by mitotic catastrophe	$5 \times 10^{-4}$
	$\eta _{\text {R}}$	Clearance rate of cells killed by RT	$5 \times 10^{-5}$
High HT	${\widetilde{\beta }}$	Rate of HT-induced damage	$[10^{-3}, 10^{-2}]$
	$\kappa _0$	Rate of angiogenesis	$10^{-7}$
	$\eta _{\text {H}}$	Clearance rate of cells killed by HT	$5 \times 10^{-5}$
	$\mu _{\Lambda }$	HT weighing factor	2.5
	$k_1$	Repair protein inactivation rate	$100 {\widetilde{\beta }}$
	$k_2$	Repair protein reactivation rate	$10^{-3}$

*Defining the Model Parameters*. We fix our model parameters at the values and ranges stated in Table 2. Parameters relating to tumour growth and RT response are fixed at the values used in Colson et al. (2022) and Colson et al. (2023), respectively. For parameters relating to high HT, the values of $\kappa _0, \,\eta _{\text {H}},\, \mu _{\Lambda }, \, k_1$ and $k_2$ are based on literature estimates (Jafari Nivlouei et al. 2021; Weyland et al. 2020). We also estimate a physically realistic range for ${\widetilde{\beta }}$ by combining values from the literature (Wright 2013) with preliminary numerical simulations (results not shown).

*Quantities of Interest*. For each simulation, we record ${\bar{T}}, \, {\bar{T}}_{\text {S}},\, {\bar{T}}_{\text {R}}, \, {\bar{T}}_{\text {H}}$, ${\bar{V}}$ and ${\bar{c}}$, the mean values of the dependent variables during the last week of treatment. We then define the percent change in (mean) viable tumour cell, total cell and vascular volumes and (mean) oxygen concentration between the start and the end of treatment as follows:

23 $$\begin{aligned} \begin{aligned} \Delta ^X_{\text{ viable }}&= 100 \times \frac{({\bar{T}} + {\bar{T}}_{\text{ S }})-T^*}{T^*}, \quad \Delta ^X_{\text{ total }} = 100 \times \frac{{\bar{\Sigma }} -\Sigma _0}{\Sigma _0}, \\ \Delta ^X_{\text{ V }}&= 100 \times \frac{{\bar{V}} -V(0)}{V(0)}, \quad \Delta ^X_{\text{ c }} = 100 \times \frac{{\bar{c}}-c^*}{c^*}, \end{aligned} \end{aligned}$$

where $X = {\text {R}}$ for RT, $X = {\text {H}}$ for HT, $X = {\text {R}}+{\text {H}}$ for ${\text {RT}}+{\text {HT}}$, $\Sigma _0 = T^*+V(0)$ and ${\bar{\Sigma }} = {\bar{T}} + {\bar{T}}_{\text {S}} + {\bar{T}}_{\text {R}} + {\bar{T}}_{\text {H}} + {\bar{V}}$.

**Table 3:** For each virtual population and RT (HT) schedule, we define $q_{10}$, $q_{37.5}$ and $q_{62.5}$ to be the $10\%$, $37.5\%$ and $62.5\%$ percentiles, respectively, of the distribution of $\Delta ^{{\text {R}}}_{\text {viable}} + \Delta ^{{\text {R}}}_{\text {total}}$ ($\Delta ^{{\text {H}}}_{\text {viable}} + \Delta ^{{\text {H}}}_{\text {total}}$). These percentiles qualitatively define limited, moderate, positive and strongly positive tumour responses to RT (HT)

$$\Delta ^{X}_{\text {viable}} + \Delta ^{X}_{\text {total}}$$	$$[q_{62.5},0)$$	$$[q_{37.5}, q_{62.5})$$	$$[q_{10},q_{37.5})$$	$$(-\infty ,q_{10})$$
Response	Limited	Moderate	Positive	Strongly positive

For RT ($X=R$) and HT ($X=H$) alone, we evaluate treatment efficacy based on the value of ${\Delta ^{X}_{\text {viable}} + \Delta ^{X}_{\text {total}}}$, with large negative values implying effective treatments. For each virtual cohort and treatment protocol, we use the distribution of $\Delta ^{X}_{\text {viable}} + \Delta ^{X}_{\text {total}}$ to distinguish between tumours with limited, moderate, positive and strongly positive responses using the criteria in Table 3. Further, if $\max {(\Delta ^{X}_{\text {viable}} + \Delta ^{X}_{\text {total}}, \Delta ^{X}_{\text {V}}}) > 0$, then treatment *X* is deemed deleterious.

### Comparing the Efficacy of RT, HT and RT+HT Treatments

**Fig. 5:**
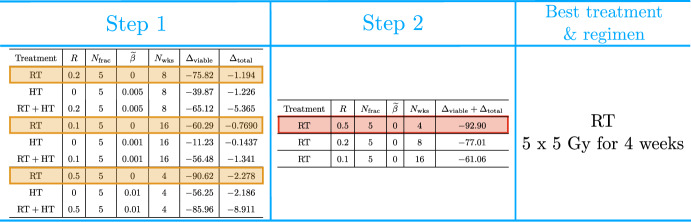
Schematic showing our two-step process for comparing RT, HT and ${\text {RT}}+{\text {HT}}$ treatments on a toy dataset generated by taking a subset of the simulation results for a particular tumour in the SL cohort. The orange boxes highlight the best treatment, for comparable RT, HT and ${\text {RT}}+{\text {HT}}$ schedules, and the red box highlights the best treatment and dosing regimen for this tumour.

For each virtual tumour, we compare RT, HT and ${\text {RT}}+{\text {HT}}$ treatments as follows (see Fig. 5 for an implementation of our method on a toy dataset). First, we probe interactions between RT and HT by comparing the three treatments for fixed RT and HT dosing schedules (e.g., Fig. 4). Given each pair of RT and HT protocols, we determine the most effective treatment (RT, HT or ${\text {RT}}+{\text {HT}}$) using the following rules:

1. Treatment $X\in \{R,H,R+H\}$ is ineffective and excluded from consideration if
  24 $$\begin{aligned} \max {\big \lbrace \Delta ^X_{\text {viable}},\; \Delta ^X_{\text {total}}, \; \Delta ^X_{\text {V}} \big \rbrace } > 0. \end{aligned}$$
2. Combined treatment is more effective than treatment $X \in \{R,H\}$ if
  25 $$\begin{aligned} \max {\left( \Delta ^{\text {R+H}}_{\text {viable}} - \Delta ^{X}_{\text {viable}}, \Delta ^{\text {R+H}}_{\text {total}}-\Delta ^{X}_{\text {total}} \right) } \le -10, \quad \Delta ^{\text {R+H}}_{\text {V}} < \Delta ^{X}_{\text {V}}. \end{aligned}$$
3. HT is more effective than RT (and conversely) if
  26 $$\begin{aligned} \Delta ^{\text {H}}_{\text {viable}}+ \Delta ^{\text {H}}_{\text {total}} < \Delta ^{\text {R}}_{\text {viable}} + \Delta ^{\text {R}}_{\text {total}}. \end{aligned}$$

Rule 1 selects for treatments with positive tumour responses. Rule 2 requires combined treatment to ‘significantly’ outperform the better of RT or HT to be considered more effective. Here, the reductions in viable and total tumour volumes must be at least $10 \%$ larger than for RT and HT alone. A milder condition is imposed on the reduction in vascular volume as it typically has a smaller impact on tumour burden (since $V \ll \Sigma$). Rule 3 implies that HT is more effective than RT if it leads to a greater combined reduction in viable and total volumes (and conversely). Note that, for effective HT and ${\text {RT}}+{\text {HT}}$, we have $\Delta ^{\text {R+H}}_{\text {V}} < \Delta ^{\text {R}}_{\text {V}}$ and $\Delta ^{\text {H}}_{\text {V}} < \Delta ^{\text {R}}_{\text {V}}$ since $\Delta ^{\text {R}}_{\text {V}} = 0$.

After comparing the three treatments for fixed RT and HT protocols, we rank the most effective treatments and associated protocols found in this first step from smallest to largest values of ${(\Delta ^X _{\text{ viable }} + \Delta ^X _{\text{ total }})}$. After adjusting the ranking to ensure Rule 2 is satisfied, the best treatment and dosing regimen among those considered is ranked first.

## Review of Previous Results: Tumour Responses to RT

**Fig. 6:**
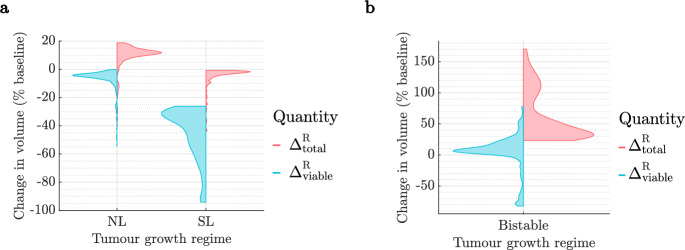
Violin plots of the distributions of $\Delta ^{\text {R}}_{\text {viable}}$ and $\Delta ^{\text {R}}_{\text {total}}$ following conventional RT for tumours in the **a** NL and SL cohorts, and **b** BS cohort. SL tumours respond well to RT as they experience reductions in viable and total tumour volumes, while NL tumours have more limited responses, with smaller reductions in viable volume and increases in total volume. In the BS cohort, RT has a deleterious effect. Figures **a** and **b** were reproduced from Colson et al. (2023).

We first summarise previous findings reported in Colson et al. (2023) regarding short-term responses to RT. As shown in Fig. 6, we distinguished between the response of tumours in the monostable (NL, SL) and bistable regimes. Although SL tumours typically experience larger reductions in viable and total volumes, NL and SL tumours respond positively to RT. By contrast, tumours in the BS cohort respond poorly to RT as they may switch from a NL to SL steady state and experience significant increases in viable and/or total volumes.

**Fig. 7:**
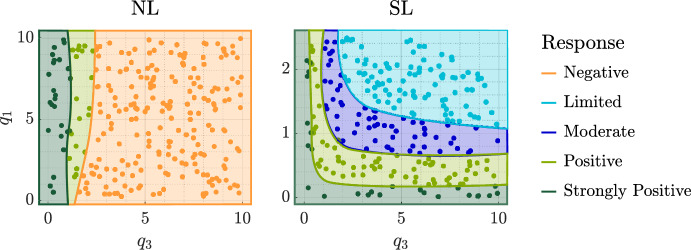
For the NL and SL cohorts, we show how the efficacy of RT depends on the values of $q_1$ and $q_3$. We stratify tumour responses into five classes: negative, limited, moderate, positive and strongly positive, as described in Sect. 3.3.

Figure 6 also illustrates the large intra-cohort variability we observed in tumour responses to RT. We explained this variability by assessing how the values of the oxygen consumption rates for maintenance and proliferation, $q_1$ and $q_3$, impact RT responses in each regime, across the RT schedules considered. Figure 7 shows that NL tumours only respond positively to RT when $q_3$ is low. While poorly oxygenated NL tumours experience little RT cell kill, low values of $q_3$ enable a positive RT response by limiting tumour regrowth between fractions. Further, the RT response of SL tumours improves as $q_1$ and/or $q_3$ decrease. This is because low $q_1$ implies high oxygen levels and, thus, high RT cell kill rates, while low $q_3$ means limited tumour regrowth, as for NL tumours. Since all tumours in the BS cohort respond poorly to RT, we do not discuss the influence of $q_1$ and $q_3$ on their RT response here; we refer the interested reader to Colson et al. (2023).

Finally, we investigated the impact of varying the radiation dose rate, *R*, and the number of weekly fractions, $N_{\text {frac}}$, (for a fixed total dose) on RT response. We found that tumours in the SL cohort respond best when larger doses are applied at higher frequency, while tumours in the NL and BS cohorts have an enhanced response when smaller doses are applied at lower frequency.

## Investigating Tumour Responses to High HT

We now assess tumour responses to high HT in the NL, SL and BS virtual cohorts using the methodology in Colson et al. (2023) to characterise tumour responses to RT. For fixed HT schedules, we consider the distributions of $\Delta ^\text {H}_\text {viable}$, $\Delta ^\text {H}_\text {total}$ and $\Delta ^\text {H}_\text {V}$ and identify the typical (i.e., average) response in each virtual cohort. Where there is high intra-cohort variability in HT responses, we also determine the influence of the values of the oxygen consumption rates, $q_1$ and $q_3$. In Sect. 5.1, we summarise our findings for short-term tumour responses to “conventional” high HT ($1 \times {\widetilde{\beta }} = 0.005$ for $N_{\text {wks}} = 8$ weeks). We then investigate the impact of varying the HT dose ${\widetilde{\beta }} \in [0.001,0.01]$ and the treatment duration $N_{\text {wks}} \le \beta _\text {max}/{\widetilde{\beta }}$ in Sect. 5.2.

### Short-Term Tumour Responses to Conventional High HT

**Fig. 8:**
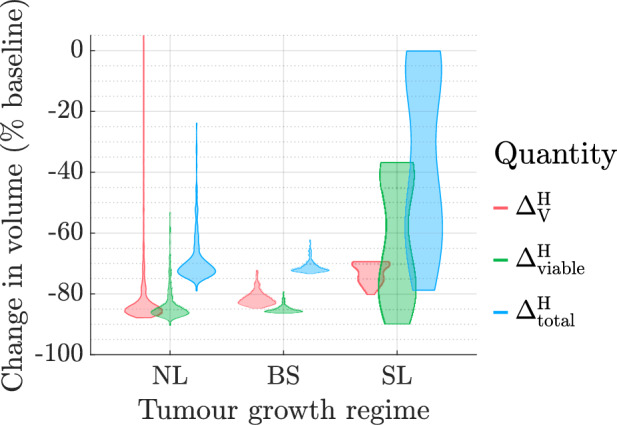
Violin plots of the distributions of $\Delta ^\text {H}_\text {V}$, $\Delta ^\text {H}_\text {viable}$ and $\Delta ^\text {H}_\text {total}$ following conventional high HT in the NL, SL and BS cohorts. All but one NL tumour respond positively to treatment, with typically greater reductions in tumour cell and vascular volumes in the NL and BS cohorts than in the SL cohort.

Figure 8 shows that all tumours respond positively to conventional high HT (i.e., $\max {(\Delta ^\text {H}_\text {V}, \Delta ^\text {H}_\text {viable}, \Delta ^\text {H}_\text {total})} < 0$) except one NL tumour which experiences an increase in vascular volume. By contrast to the RT responses discussed in Sect. 4, tumours in the NL and BS cohorts typically respond better to HT than tumours in the SL cohort, as they experience larger reductions in tumour cell and vascular volumes. Indeed, tumour responses to HT in the SL cohort are mixed, as indicated by the bimodal distributions of $\Delta ^\text {H}_\text {V}$, $\Delta ^\text {H}_\text {viable}$ and $\Delta ^\text {H}_\text {total}$.

**Fig. 9:**
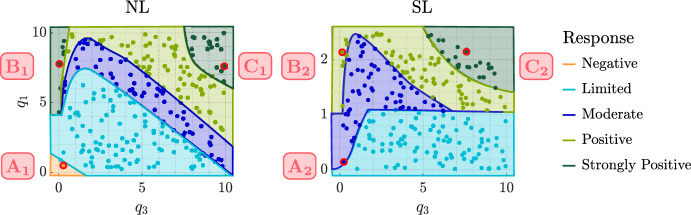
For the NL and SL cohorts, we show how the efficacy of conventional high HT depends on the values of $q_1$ and $q_3$. Tumour responses are stratified into five classes: negative, limited, moderate, positive and strongly positive, as described in Sect. 3.3. $A_1-C_1$ are representative NL tumours corresponding to $(q_3,q_1)$ sets $(2.72 \times 10^{-1}, 5.03 \times 10^{-1}), (4.01 \times 10^{-2}, 7.78)$ and (9.94, 7.60), respectively, while $A_2-C_2$ are representative SL tumours corresponding to $(q_3,q_1)$ sets $(2.10\times 10^{-1}, 1.51\times 10^{-1})$, $(1.43\times 10^{-1}, 2.14)$ and (7.61, 2.14), respectively.

To understand the high intra-cohort variability for the NL and SL populations, Fig. 9 shows how responses to high HT depend on the values of $q_1$ and $q_3$. Tumour responses in the NL cohort improve as the values of $q_1$ and/or $q_3$ increase, with the single negative responder characterised by low $q_1$ and $q_3$, and the best responders by high $q_1$, and either low $q_3$ or high $q_3$. In the SL cohort, the value of $q_1$ has a greater influence on tumour responses to HT than the value of $q_3$: given $q_3$, the value of $q_1$ must be sufficiently high to elicit a positive response. In particular, the worst responders are characterised by low values of $q_1$ and high values of $q_3$. As for the best NL responders, the best SL responders are characterised by high $q_1$, and either low $q_3$ or high $q_3$. We explain these results in more detail below.

**Fig. 10:**
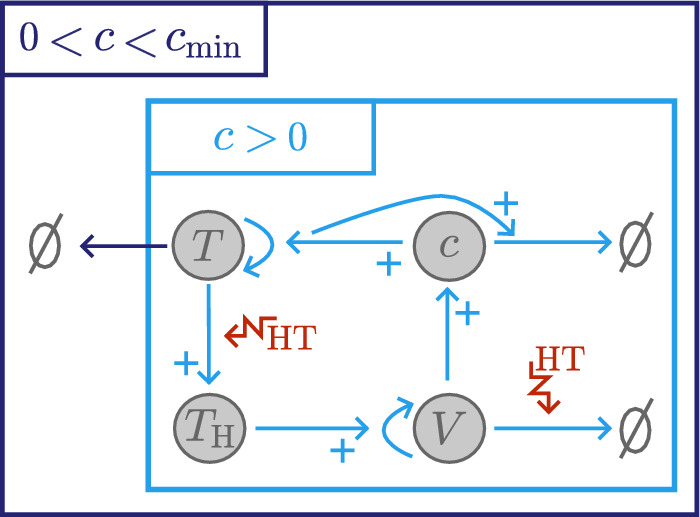
Schematic showing the relationships between the undamaged tumour volume, *T*, HT-damaged tumour volume, $T_\textrm{H}$, vascular volume, *V*, and oxygen concentration, *c*, in model (14)–(19). When HT is applied, *T* cells are lethally damaged, becoming ${T}_{\textrm{H}}$ cells. HT also kills *V*, while $T_{\text {H}}$ cells induce vascular regrowth via angiogenesis; the balance of these two processes determines whether *V* decreases or increases during treatment. Increases (decreases) in *V* lead to an increase (decrease) in the supply of *c* to the tumour and, thus, an increase (decrease) in tumour cell proliferation and decrease (increase) in hypoxic cell death. The balance between changes in oxygen supply by *V* and oxygen consumption by *T* determine whether *c* increases or decreases during treatment, which, in turn, determines the extent of regrowth of *T* during treatment.

**Fig. 11:**
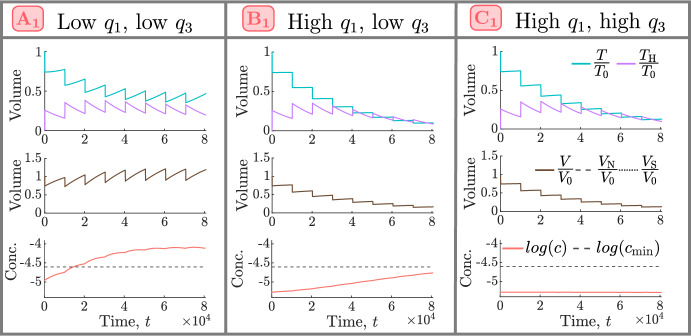
For high HT (${\widetilde{\beta }} = 0.005$), we solve Eqs. (14)–(19) for $t \in (0,8.064 \times 10^4]$ subject to initial conditions (22). We fix $V(0) = 0.0005$ and $(q_3,q_1)$ as indicated by the points $A_1$-$C_1$ in Fig. 9. Low values of $q_1$ and $q_3$ ($A_1$) lead to worse treatment outcomes than high values of $q_1$ and/or $q_3$ ($B_1$, $C_1$) as they are associated with greater accumulation of dead material, HT-induced angiogenesis and tumour cell regrowth.

**Fig. 12:**
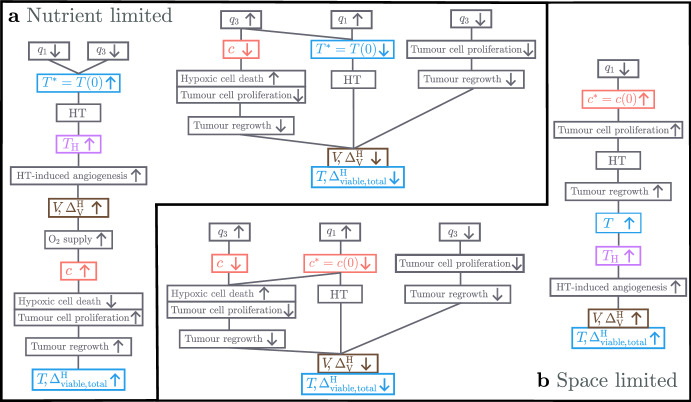
Schematics showing the biological processes that explain positive and negative responses to high HT in the **a** NL and **b** SL cohorts and how they depend on the values of $q_1$ and $q_3$.

*Tumour Responses in the NL and BS Cohorts*. Recalling Fig. 3, we see that, in the NL cohort, the steady state tumour volume in the absence of treatment, $T^* = T(0)$, increases significantly (up to 10-fold) as $q_1$ and/or $q_3$ decrease. We consider the relationships between the model’s variables (*T*, $T_{\text {H}}$, *V* and *c*) under HT, depicted in the schematic in Fig. 10, and the response of tumour $A_1$ shown in Fig. 11. We deduce that tumours in the NL cohort with low values of $q_1$ and $q_3$ have a limited, or negative, response to HT because they accumulate more dead cells, and, hence, experience a stronger angiogenic response, ultimately increasing tumour oxygenation above the hypoxic threshold and enabling more rapid tumour regrowth between HT fractions (see Fig. 12a).

Tumours in the NL cohort with higher values of $q_1$ and/or $q_3$ have smaller values of $T^* = T(0)$. They, thus, have a positive response to HT as they experience a large reduction in vascular volume, which contributes to large reductions in viable and total volumes (see the responses of tumours $B_1$ and $C_1$ in Fig. 11). Further, as shown in Fig. 12a, high values of $q_1$, combined with low or high values of $q_3$, characterise the best NL responders because tumour regrowth between HT fractions is limited due to (i) low tumour cell proliferation rates when $q_3$ is low (e.g., $B_1$) and (ii) maintenance of low oxygen levels when $q_3$ is high (e.g., $C_1$).

To understand high HT response in the BS cohort, recall that these tumours are at their NL steady state when HT is applied. Since tumours in this cohort have high values of $q_3$ (see Fig. 3), they respond to high HT similarly to NL tumours with high values of $q_3$ (e.g., tumour $C_1$ in Fig. 11), and their strong HT response follows similarly.

**Fig. 13:**
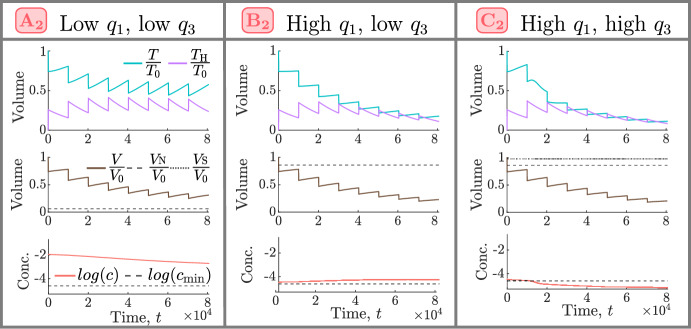
For high HT (${\widetilde{\beta }} = 0.005$), we solve Eqs. (14)–(19) for $t \in (0,8.064 \times 10^4]$ subject to initial conditions (22). We fix $V(0) = 0.005$ and $(q_3,q_1)$ as indicated by the points $A_2 - C_2$ in Fig. 9. Low values of $q_1$ ($A_2$) lead to worse outcomes than high values of $q_1$ ($B_2$, $C_2$) as higher oxygen levels promote greater tumour regrowth and dead cell accumulation.

*Tumour Responses in the SL Cohort.* Fig. 13 shows that SL tumours with low values of $q_1$ (e.g., $A_2$) have higher oxygen levels at baseline and throughout treatment than SL tumours with high values of $q_1$ (e.g., $B_2$, $C_2$). Consequently, tumours with low $q_1$ proliferate faster and experience larger tumour regrowth between fractions than tumours with high $q_1$. As shown in Fig. 12b, this leads to smaller reductions in tumour and vascular volumes and explains why tumours with low $q_1$ have a limited response to high HT.

Pre-treatment oxygen levels of SL tumours with high values of $q_1$ are close to the hypoxic threshold, $c_{\text {min}}$. Consequently, when $q_3$ is low (e.g., $B_2$), the proliferation rate is also low and limited tumour regrowth between HT fractions ensures a sustained reduction in viable and total cell volumes. When $q_3$ is large (e.g., $C_2$), oxygen levels may become hypoxic during treatment, which increases cell death and decreases tumour cell proliferation between HT fractions, ensuring even larger reductions in viable and total cell volumes, despite a high value of $q_3$.

Overall, as shown in Fig. 12b, limited tumour cell proliferation (low and high $q_3$) and hypoxic cell death (high $q_3$) can contribute to the increased efficacy of HT for SL tumours with high $q_1$. We note that these processes are similar to those also identified for NL tumours with high $q_1$.

### The Influence of High HT Dosing Regimen

**Fig. 14:**
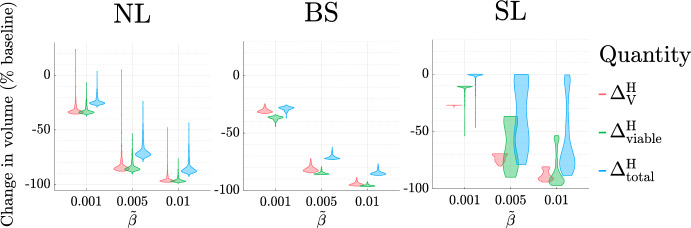
For the NL, BS and SL virtual cohorts, we show how the distributions of $\Delta ^{\text {H}}_{\text {V}}$, $\Delta ^{\text {H}}_{\text {viable}}$ and $\Delta ^{\text {H}}_{\text {total}}$ change as ${\widetilde{\beta }} \in \{0.001,0.005, 0.01 \}$ varies (for fixed $N_{\text {wks}}=8$). The values of $\Delta ^{\text {H}}_{\text {V}}$, $\Delta ^{\text {H}}_{\text {viable}}$ and $\Delta ^{\text {H}}_{\text {total}}$ typically decrease as ${\widetilde{\beta }}$ increases.

*The Efficacy of High HT Increases with the Total Thermal Dose*, ${\beta }_{\text {total}}$. Figure 14 shows that increasing the value of the fraction dose, ${\widetilde{\beta }}$, for a fixed treatment duration ($N_{\text {wks}}=8$ weeks), enhances tumour responses to high HT in each virtual cohort. For the NL and BS cohorts, larger values of ${\widetilde{\beta }}$ lead to greater reductions in the tumour cell and vascular volumes. For the NL cohort, if ${\widetilde{\beta }} \ge 0.007$, then any tumour whose vascular volume increases at low doses (e.g., tumour $A_1$ in Fig. 11 for ${\widetilde{\beta }} =0.005$) responds positively to treatment. For the SL cohort, increasing ${\widetilde{\beta }}$ markedly improves the treatment response, with most tumours responding similarly to those in the NL and BS cohorts.

We observe similar behaviour as the treatment duration, $N_{\text {wks}}$, increases for fixed ${\widetilde{\beta }}$ (results not shown). Since ${\beta }_{\text {total}}$ increases as ${\widetilde{\beta }}$ and $N_{\text {wks}}$ increase, we conclude that maximising the total thermal dose leads to the best treatment outcome.

**Fig. 15:**
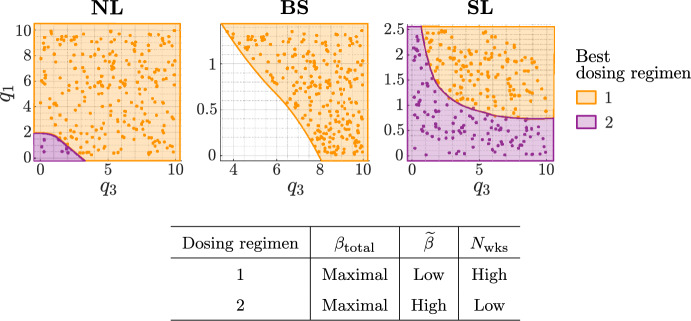
Scatter plots showing the HT dosing regimen (defined in the table) which maximises $|\Delta ^{\text {H}}_{\text {viable}}+\Delta ^{\text {H}}_{\text {total}}|$ for each $(q_3,q_1)$ pair used to generate the NL, BS and SL virtual cohorts. The best dosing regimen depends on the efficacy of high HT: when HT response is weaker, larger HT fractions applied over a shorter time period typically maximise treatment efficacy (purple), whereas, when HT response is stronger, lower HT fractions applied over a longer time period are typically best (orange).

*The Best High HT Dosing Regimen Depends on the Values of *$q_1$ *and* $q_3$. We have found that increasing the total thermal dose enhances treatment outcome. We now determine the dose and treatment duration that maximise treatment efficacy (by minimising $\Delta ^{\text {H}}_{\text {viable}} + \Delta ^{\text {H}}_{\text {total}}$). Figure 15 shows the two best performing dosing protocols across the virtual cohorts. Consistent with the results in Fig. 14, the best protocol maximises the total thermal dose for all tumours. It also depends on the growth regime and the values of $q_1$ and $q_3$.

**Fig. 16:**
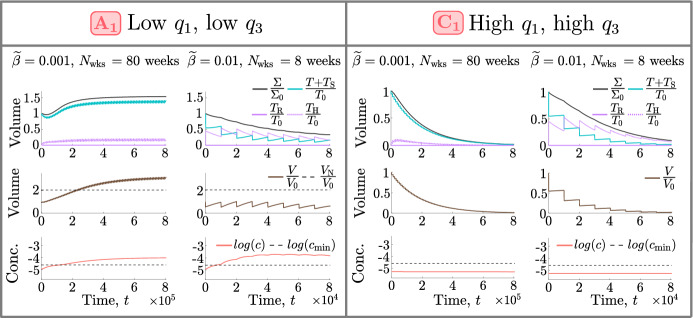
We solve Eqs. (14)–(19) subject to initial conditions (22). We fix ${V(0)= 0.0005}$, and $(q_3,q_1)$ as indicated by points $A_1$ and $C_1$ in Fig. 9. In both cases, we fix the thermal dose (left) ${\widetilde{\beta }} = 0.001$ or (right) ${\widetilde{\beta }} = 0.01$, and simulate high HT for (left) 80 weeks or (right) 8 weeks. A larger thermal dose is necessary for $A_1$ to achieve a sustained reduction in tumour burden. In contrast, a lower thermal dose applied over a longer period yields a more gradual, but larger, reduction in tumour burden for $C_1$.

Tumours which respond best to dosing regimen 1 typically respond better to high HT than tumours which respond best to dosing regimen 2 (see Sect. 5.1). For the former, a larger number of low-dose HT fractions enables a more gradual, but larger, reduction in tumour burden (e.g., see the responses of NL tumour $C_1$ in Fig. 16). For the latter, low-dose HT may be deleterious as tumour regrowth may exceed cell death, while high-dose HT increases cell death markedly, enabling large reductions in vascular and viable volumes (e.g., see the responses of NL tumour $A_1$ in Fig. 16).

We conclude this section by noting that the results described above hold for a fixed rate of HT-induced angiogenesis ($\kappa _0 = 10^{-7}$). Letting $\kappa _0 \in \{10^{-8}, 10^{-7},10^{-6} \}$ vary (results not shown for brevity; see (Colson 2023)), we found that high HT can be extremely effective for tumours in all regimes if their angiogenic response is weak ($\kappa _0 \le 10^{-7}$). If the angiogenic response is strong ($\kappa _0 > 10^{-7}$), then most tumours in the NL and BS cohorts respond poorly to treatment, while all tumours in the SL cohort have a reduced, but still positive, response. Therefore, the high HT responses predicted by our model depend critically on the value of $\kappa _0$. However, we assumed that only high HT affects the vasculature and, in particular, we neglected angiogenesis and vascular remodelling in the absence of treatment and in response to RT. Due to this substantial simplification of tumour vascular dynamics, the influence of HT-induced angiogenesis on tumour responses to high HT should be interpreted with caution. We, thus, leave assessing the impact of angiogenesis on high HT and combined treatment as future work (see Sect. 7) and we fix $\kappa _0 = 10^{-7}$ for the remainder of this paper. Also as a result of our simplifying assumptions about tumour angiogenesis, the short-term tumour responses to high HT described in this section determine long-term tumour responses (results not shown for brevity; see (Colson 2023)), i.e., the best (worst) responders in the short-term correspond to the best (worst) responders in the long-term. As such, the long-time dynamics of this model should also be interpreted with caution, which is why we focus on short-term responses to HT and ${\text {RT}}+{\text {HT}}$ in this paper.

## Investigating Tumour Responses to RT+HT

We now build on our understanding of tumour responses to RT (Sect. 4) and high HT (Sect. 5) to explain tumour responses to their combination. We determine the tumour-specific treatment and dosing regimen that maximise treatment outcome, as defined in Sect. 3.4, across each virtual cohort. Recall here that combined treatment is only considered best if it elicits a reduction in viable and total volumes that is at least $10\%$ larger than RT and HT alone. Our results for the SL cohort are sufficient to describe the key trends relating to which tumours respond best to RT, HT or ${\text {RT}}+{\text {HT}}$. Therefore, for brevity, the corresponding results for the NL and BS cohorts are summarised below.

**Fig. 17:**
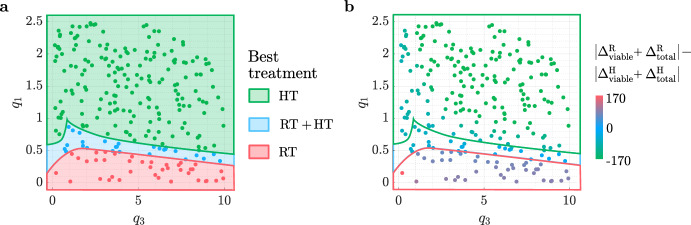
For the $(q_3,q_1)$ pairs used to generate the SL virtual cohort, scatter plot **a** shows the best treatment and scatter plot **b** shows the values of $|\Delta ^{\text {R}}_{\text {total}} + \Delta ^{\text {R}}_{\text {viable}} | - |\Delta ^{\text {H}}_{\text {total}} + \Delta ^{\text {H}}_{\text {viable}} |$ for comparable RT and HT protocols (recall Fig. 4) corresponding to the optimal schedules in Fig. 18. Tumours that respond much better to RT than HT typically respond best to RT, and vice versa, while tumours with comparable responses to RT and HT alone respond best to combined treatment.

**Fig. 18:**
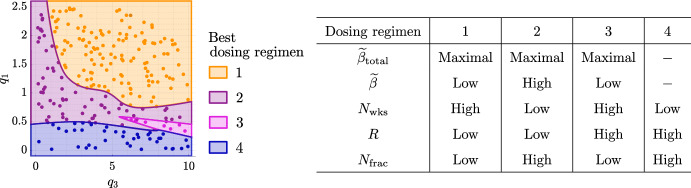
Scatter plots showing the best dosing regimen (defined in the table) for the $(q_3,q_1)$ pairs used to generate the SL virtual cohort. When HT alone is best (see Fig. 17), a longer type 1 regimen with lower HT fractions is typically recommended for tumours that have a stronger response to HT alone and weaker response to RT alone. A shorter type 2 regimen with higher HT fractions is recommended for tumours with a weaker response to HT and stronger response to RT. When ${\text {RT}}+{\text {HT}}$ is best (see Fig. 17), tumours with low values of $q_3$ respond best to the type 2 regimen which includes lower RT fractions, while tumours with high values of $q_3$ respond best to a longer type 3 regimen combining low HT fractions and high RT fractions. When RT alone is best (see Fig. 17), high RT fractions applied at high frequency maximise treatment efficacy.

**Fig. 19:**
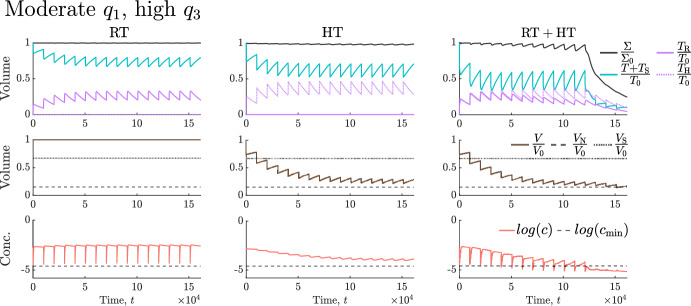
For RT ($R=0.5$, $N_{\text {frac}} = 1$) and high HT (${\widetilde{\beta }}=0.005$) schedules, alone and combined, we solve Eqs. (14)–(19) for $t \in (0,1.6128 \times 10^5]$ subject to initial conditions (22). We set $(V (0), q _3, q_1) = (0.005, 8.81, 3.74 \times 10^{-1})$. This SL tumour has a limited response to high HT and a positive response to RT (compared to the rest of the SL cohort). Combined treatment is most effective as RT and HT act synergistically, with RT significantly enhancing HT effects.

**Fig. 20:**
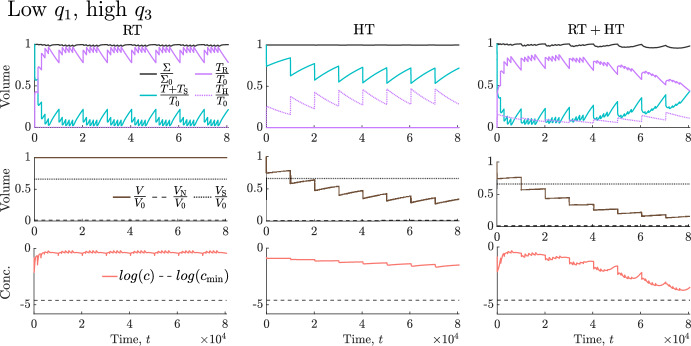
For conventional RT and HT schedules, alone and combined, we solve Eqs. (14)–(19) for $t \in (0,8.064 \times 10^4]$ subject to initial conditions (22). We set $(V (0), q_3 , q_1) = (0.005, 9.53, 3.21 \times 10^{-2})$. This SL tumour responds positively to RT, but has a limited response to high HT. During combined treatment, HT-induced vascular damage reduces oxygen levels and RT cell kill rates. Combined with fast tumour regrowth between fractions, this slows the reduction in viable volume, enabling net growth of the viable volume. As a result, RT alone is the best treatment.

In Fig. 17a, the SL cohort splits into three groups based on which treatment is most effective. Comparing tumour responses to RT and HT alone in these three groups for the optimal dosing schedules (see Fig. 18), Fig. 17b shows that tumours with a comparable response to RT and HT benefit most from combined treatment as RT and HT combine synergistically (e.g., see Fig. 19 in Appendix A). By contrast, tumours with a stronger response to RT than HT respond best to RT (and conversely) as combined treatment leads to either a negligible improvement in outcome or a worse outcome (e.g., see Fig. 20 in Appendix A).

In terms of the most effective dosing regimen, Fig. 18 identifies four optimal schedules. Dosing regimens 1 and 2 are analogous to those found for HT alone (see Fig. 15): (1) long treatments with low thermal doses per fraction and, for ${\text {RT}}+{\text {HT}}$, low RT doses applied at low frequency; (2) short treatments with high thermal doses per fraction and, for ${\text {RT}}+{\text {HT}}$, low RT doses applied at high frequency. When HT alone is optimal, the best regimens are defined in Fig. 15. When ${\text {RT}}+{\text {HT}}$ is optimal, there are two possibilities. First, the best dosing regimen for combined treatment is analogous to the best dosing regimen for HT alone; this is the case for SL tumours with low values of $q_3$. Second, SL tumours may respond best to dosing regimen 3, which combines low HT doses with high RT doses applied at low frequency. This regimen maximises the total thermal dose and the intensity of each RT fraction. Tumours which respond best to regimen 3 exhibit strong synergistic interactions between HT and RT (e.g., see Fig. 19 in Appendix A). Lastly, dosing regimen 4 is tailored to tumours that respond best to RT alone (as defined in Sect. 4).

Consistent with the results for the SL cohort, most of the NL cohort and all of the BS cohort respond best to HT alone, since HT is significantly more effective than RT for these tumours. The exception is one NL tumour which has a limited response to HT, but strongly positive response to RT; in this case, the treatments act synergistically and combined treatment is best. Dosing regimens 1 and 2 defined in Fig. 18 for combined treatment (and Fig. 15 for HT alone) maximise treatment outcomes in the NL and BS cohorts. The best dosing regimen for each tumour in Fig. 15 remains the same even when RT+HT is optimal.

## Discussion

Hyperthermia (HT) is a promising candidate for improving tumour responses to radiotherapy (RT). However, their combined use in the clinic has been limited by incomplete understanding of their potential synergistic interactions (Behrouzkia et al. 2016) and inconclusive results from clinical trials (Cihoric et al. 2015; Datta et al. 2015). In this paper, we used a mathematical approach to investigate how nutrient and space limited mechanisms of growth control may influence tumour responses to HT applied alone and in combination with RT. Our aim was to shed light on the interactions between RT and HT, characterise tumours that may respond best to RT, HT or combined treatment, and identify recommended dosing regimens in each case.

We extended an existing model of tumour growth and RT response, which distinguishes between nutrient and space limited growth (Colson et al. 2022, 2023), to include tumour responses to mild and high HT. We found that mild HT has a negligible effect when applied alone and does not significantly enhance tumour responses to RT when the treatments are combined. Therefore, we focussed on studying high HT. Building on our previous study of RT response (Colson et al. 2023), we systematically investigated high HT alone and combined with RT, and produced testable predictions relating to how RT and high HT should be combined.

Assuming a fixed HT-induced angiogenic rate $\kappa _0 = 10^{-7}$, we found that most tumours respond better to combined treatment than RT alone, the exception being well-oxygenated space limited (SL) tumours that have low values of the oxygen consumption rate for maintenance, $q_1$. For these tumours, combined treatment either worsens treatment outcome as a smaller reduction in viable volume than RT alone is achieved, or elicits a modest ($< 10\%$) further reduction in viable volume compared to RT alone. Analogously, tumours which have a stronger response to high HT than to RT do not benefit from their combination and high HT is the recommended treatment. Therefore, high HT and RT typically have significant synergistic interactions for tumours with a comparable response to RT and HT alone.

Accordingly, most of the nutrient limited (NL) cohort and all of the bistable (BS) cohort respond best to high HT alone. By contrast, the SL cohort is split between tumours which respond best to RT, high HT and combined treatment. Our model thus supports clinical evidence suggesting that not all tumours will respond better to combined treatment than RT alone (Datta et al. 2015) and provides insight into why this might be (i.e., recall SL tumours with low $q_1$). Our model also reveals which tumours may respond best to high HT alone (i.e., NL tumours with high values of $q_1$ and/or high values of the oxygen consumption rate for proliferation, $q_3$, BS tumours and SL tumours with high values of $q_1$).

We identified four dosing regimens that can maximise the reduction in tumour burden:

1. Long HT treatments with low thermal doses per fraction;
2. Short HT (and ${\text {RT}}+{\text {HT}}$) treatments with high thermal doses per fraction (and low RT fraction doses applied at high frequency);
3. Long ${\text {RT}}+{\text {HT}}$ treatments with low thermal doses per fraction and high RT fraction doses applied at low frequency;
4. Short RT treatments with high fraction doses and frequency.

The efficacy of HT is maximised with a gradual thermal exposure (i.e., regimen 1) for tumours which have a strong, positive response to high HT, while tumours with a more limited response to high HT respond best to a faster, greater thermal exposure during HT (i.e., regimen 2). For tumours which respond best to ${\text {RT}}+{\text {HT}}$, the optimal schedule is either analogous to that for high HT alone, with the addition of low RT exposure (i.e., regimen 2), or consists of a gradual thermal exposure combined with a higher RT exposure (i.e., dosing regimen 3). This distinction reflects stronger (regimen 2 is best) vs. weaker (regimen 3 is best) responses to high HT alone. The efficacy of RT alone is maximised for SL tumours using large fractions at high frequency (i.e. regimen 4), as established in Colson et al. (2023).

Cases for which high HT alone is preferred are of particular interest since HT is typically viewed as an adjuvant treatment, rather than a primary treatment (Behrouzkia et al. 2016). This is because the few clinical trials investigating tumour responses to high HT alone suggest that its efficacy is limited (Meyer 1984). It would be interesting, in future work, to investigate whether the discrepancy between these clinical results and our model predictions are due to physical limitations of current treatment methods, which may fail to heat an entire tumour to the target temperature (Peeken et al. 2017), or an overestimation of the effects of high HT in our model. Using experimental and clinical data to calibrate and validate our model would be an important first step to answering this question.

To that end, we aim, in future work, to use model simulations to investigate whether early response to RT or HT alone (e.g., in the first couple of weeks of treatment) can reliably inform a tumour’s growth regime. In this case, we could test regime-specific model predictions using *in vitro* and *in vivo* data. Further, our modelling suggests that, regardless of the growth regime, a tumour with a strong response to RT or HT is unlikely to benefit significantly from combined treatment, while a tumour with more limited responses to RT and HT could respond best to their combination. These predictions will also be tested in future work.

In addition, we will aim to relax some of our model’s simplifying assumptions. To limit model complexity, we neglected the co-evolution of the vasculature and tumour cells in response to angiogenesis (Chaplain 1996; Farnsworth et al. 2014) and RT (Potiron et al. 2013; Stolz et al. 2022; Venkatesulu et al. 2018) that is observed *in vivo*. As a result, we cannot capture all pre/post-treatment vascular changes, which, given the importance of the rate of angiogenesis, $\kappa _0$, in predicting tumour responses to high HT, are likely to affect our results. It would, therefore, be important to extend the model by making the vascular volume a dynamic variable, which evolves in response to angiogenic cues (Hahnfeldt et al. 1999; Stamper et al. 2007), RT and HT. This would provide a more realistic description of vascular remodelling, and tumour responses to RT and HT.

Angiogenesis is an intrinsically spatial process, which has been well studied using a range of mathematical approaches; see the reviews by Heck et al. (2015); Scianna et al. (2013); Stepanova et al. (2024). Also, the spatio-temporal heterogeneity of intra-tumoural oxygen levels can impact tumour growth dynamics, intra-tumour phenotypic diversity, and treatment response (Celora et al. 2023; Chiari et al. 2023; Robertson-Tessi et al. 2015; Villa et al. 2021a, b). Thus, incorporating spatial structure into our model would be a final interesting direction for future investigation.

Overall, the results presented in this paper suggest that a one-size-fits-all approach to the design of combined RT and high HT treatments is not appropriate for tumours characterised by a single mechanism of growth arrest (i.e., NL and SL cohorts). Indeed, we found inter-tumour variability in terms of the most effective treatment, and also the dosing regimen that maximises treatment efficacy. By contrast, all tumours for which nutrient and space limited mechanisms of growth arrest are simultaneously active (i.e., BS cohort) respond best to the same high HT schedule.

## Data Availability

All simulated data generated and analysed as part of this study is available at: https://github.com/chloeacolson/InvestigatingTumourResponsesRadiotherapyHyperthermia.

## Appendix A: Additional Numerical Simulations

We present numerical simulations that support the results discussed in Sect. 6.

## Code Availability

MATLAB code to numerically solve the ODE model, and determine the best treatment and dosing regimen for each tumour is available at: https://github.com/chloeacolson/InvestigatingTumourResponsesRadiotherapyHyperthermia.
